# Rapidly Neutralizable and Highly Anticoagulant Thrombin-Binding DNA Aptamer Discovered by MACE SELEX

**DOI:** 10.1016/j.omtn.2019.03.002

**Published:** 2019-03-22

**Authors:** Koji Wakui, Toru Yoshitomi, Akane Yamaguchi, Maho Tsuchida, Shingo Saito, Masami Shibukawa, Hitoshi Furusho, Keitaro Yoshimoto

**Affiliations:** 1 Department of Life Sciences, Graduate School of Arts and Sciences, The University of Tokyo, 3-8-1 Komaba, Meguro, Tokyo 153-8902, Japan; 2 Graduate School of Science and Engineering, Saitama University, 255 Shimo-Okubo, Sakura-ku, Saitama 338-8570, Japan; 3 Chemical General Division, Nissan Chemical Industries, Ltd., 2-10-2 Tsuboi-nishi, Funabashi, Chiba 274-8507, Japan; 4 JST, PRESTO, The University of Tokyo, 3-8-1 Komaba, Meguro, Tokyo 153-8902, Japan

**Keywords:** aptamer, anticoagulant, thrombin, SELEX, antidote, toehold-mediated strand displacement, capillary electrophoresis

## Abstract

We present a rapidly neutralizable and highly anticoagulant thrombin-binding aptamer with a short toehold sequence, originally discovered by systematic evolution of ligands by exponential enrichment (SELEX) with microbead-assisted capillary electrophoresis (MACE). MACE is a novel CE-partitioning method for SELEX and able to separate aptamers from a library of unbound nucleic acids, where the aptamer and target complexes can be detected reliably and partitioned with high purity even in the first selection cycle. Three selection rounds of MACE-SELEX discovered several TBAs with a nanomolar affinity (K_d_ = 4.5–8.2 nM) that surpasses previously reported TBAs such as HD1, HD22, and NU172 (K_d_ = 118, 13, and 12 nM, respectively). One of the obtained aptamers, M08, showed a 10- to 20-fold longer prolonged clotting time than other anticoagulant TBAs, such as HD1, NU172, RE31, and RA36. Analyses of the aptamer and thrombin complexes using both bare and coated capillaries suggested that a large number of efficient aptamers are missed in conventional CE-SELEX because of increased interaction between the complex and the capillary. In addition, the toehold-mediated rapid antidote was designed for safe administration. The efficient aptamer and antidote system developed in the present study could serve as a new candidate for anticoagulant therapy.

## Graphical Abstract

**Figure:**
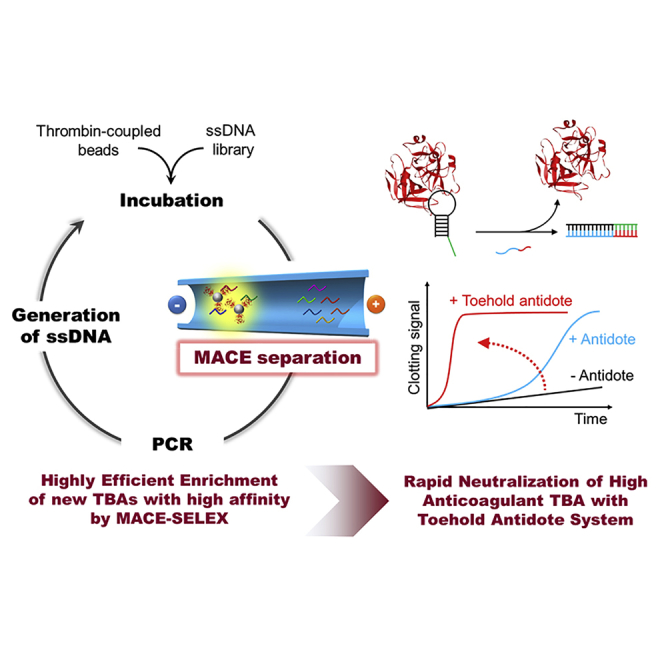

## Introduction

Anticoagulant drugs have been the mainstay for the treatment and prevention of thrombotic disorders. However, clinically used anticoagulant drugs have a risk of side effects, such as significant bleeding, that increase patient morbidity and mortality.^1^, ^2^ Hence, the lack of specific reversal agents limits their use.

DNA or RNA aptamers are single-stranded oligonucleotides with high affinity for specific targets such as ions,^3^, ^4^ small molecules,^5^, ^6^, ^7^, ^8^ peptides,^9^, ^10^, ^11^, ^12^, ^13^ proteins,^14^, ^15^, ^16^ and entire cells.^17^, ^18^, ^19^ Compared with antibodies, aptamers offer several specific advantages because of their low cost of production, ease of chemical modification, relatively small size, and lower immunogenicity.^20^, ^21^ In addition, aptamers have great benefits for antidote-mediated controllability by adding reverse complementary sequences, unlike for other types of drugs.^22^, ^23^, ^24^, ^25^ Thrombin-binding aptamers (TBAs) are important in the medical and clinical fields because thrombin is a multifunctional serine protease that plays a key role in thrombosis, homeostasis, and inflammation.^26^ So far, various aptamers to human α-thrombin, such as HD1,^15^ HD22,^27^ NU172,^28^ RE31,^29^ and RA36,^30^ have been developed. Although HD1 and NU172 have advanced to phases I and II of clinical trials, respectively, for coronary artery bypass graft surgery, no update is available regarding the current situation.^31^ On the other hand, the REG1 anticoagulant system containing the factor Ixa-binding aptamer and the complementary sequence for the antidote proceeded to a phase III clinical trial as an anticoagulant agent for percutaneous coronary intervention.^22^, ^32^ However, the efficiency of typical Watson-Crick base-pairing antidotes is not sufficient, and they require much higher doses or continuous administration to neutralize the drug activity,^22^, ^32^ resulting in an increase in cost and burden on patients. If there were aptamer-based anticoagulants with higher affinity and more efficient reversal agents, they could be promising anticoagulant systems.

Aptamers are generated by systematic evolution of ligands by exponential enrichment (SELEX) or by *in vitro* selection.^33^, ^34^ Usually, SELEX involves repeated rounds of the following steps: (1) incubation of a large random sequence library with the target, (2) partitioning of target-binding sequences, (3) amplification of the sequences by PCR, and (4) sequencing to identify sequences of aptamers. Typically, SELEX requires up to 20 selection rounds to furnish aptamers and is very laborious. To shorten the time required to obtain aptamers and to increase the efficiency of selection, a number of modifications have been developed that improve the basic procedural steps of SELEX (1,^35^, ^36^ 2,^37^, ^38^, ^39^, ^40^ 3,^41^ and 4^42^, ^43^, ^44^, ^45^). Among the aforementioned points, partitioning of the aptamer-target complexes is a particularly critical step for rapid enrichment of the aptamers in SELEX. Although capillary electrophoresis (CE)-SELEX^38^, ^39^, ^40^ so far represents the most efficient separation method, its success remains restricted by several limitations. During CE separation, the identification of the aptamer and target complexes by UV or fluorescence detection is generally difficult because of the low concentrations of aptamer and target complexes. Thus, undetected aptamer and target complexes may be collected blindly within a relatively broad collection window that may also contain low-affinity aptamers or even free oligonucleotides.^46^, ^47^ In addition, target molecules applicable to CE-SELEX are limited because a large zeta potential shift upon binding is required to separate aptamer and target complexes from free oligonucleotides.

**Figure 1:**
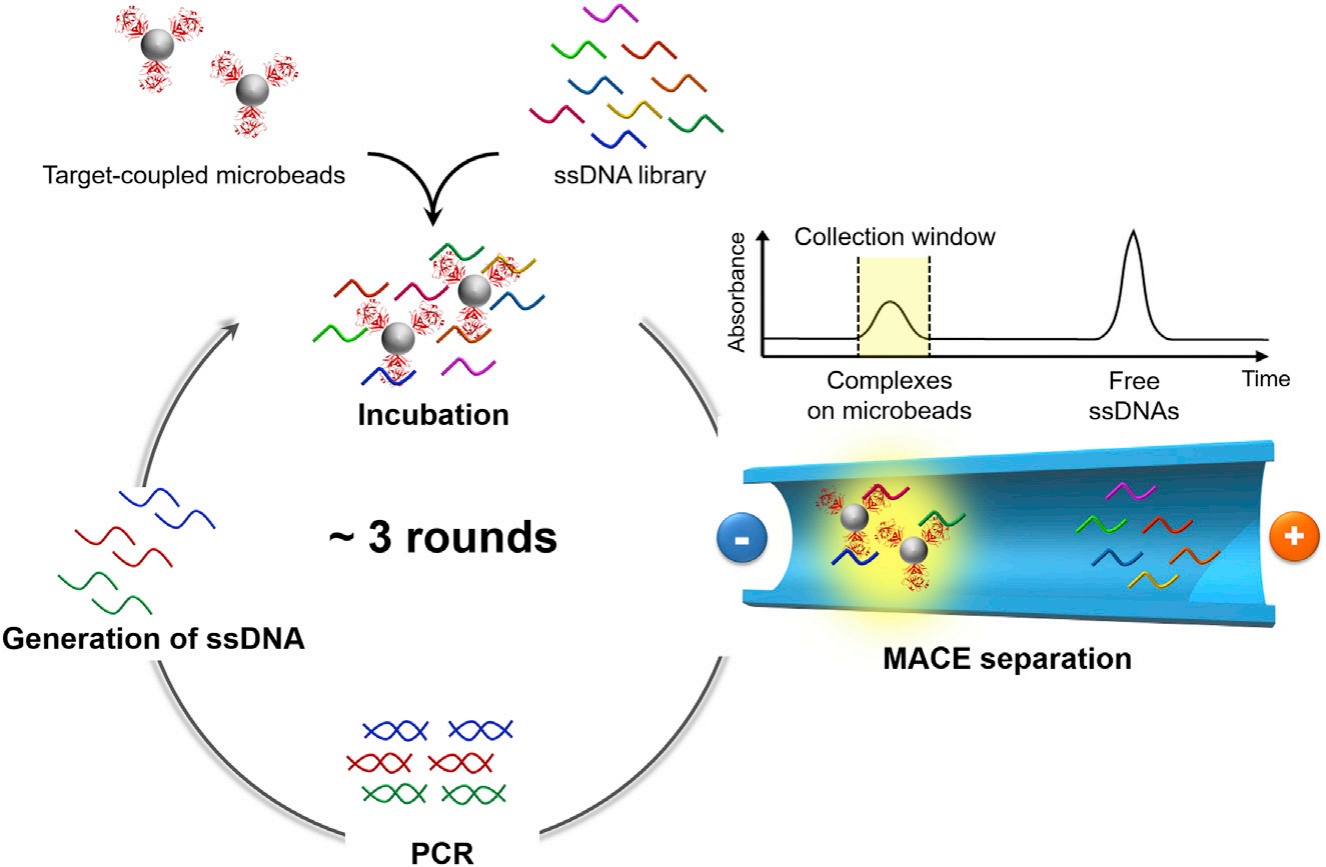
Schematic Illustration of MACE-SELEX against Thrombin in the Present Study

Here, to rapidly acquire thrombin-binding aptamer candidates with higher affinity for anticoagulant therapy, we developed a robust SELEX system with microbead-assisted CE (MACE; Figure 1). During MACE separation, an incubated mixture of target-coupled microbeads and an oligonucleotide library are directly introduced into a capillary. Because the elution time of the target-coupled microbeads is significantly different from that of the oligonucleotide library, the aptamer and target complexes can be identified by UV detection using the absorbance change that originates from the light scattering of the microbeads. Thus, the target-bound aptamers can be effectively separated and collected even in the first selection round. After three rounds of MACE-SELEX, an aptamer with 10- to 20-fold higher anticoagulant activity than reported previously for other TBAs was discovered. Additionally, utilizing toehold-mediated DNA strand displacement, we developed a rapid reversible anticoagulant system for safe administration of the discovered highly anticoagulant TBA.

## Results

### Selection by MACE-SELEX and Conventional CE-SELEX

We propose MACE-SELEX as a novel SELEX system that contains a sophisticated separation step with high sensitivity based on CE separation using target-coupled microbeads. In the present study, conventional CE-SELEX was also performed for comparison with MACE-SELEX to evaluate efficiency.

**Figure 2:**
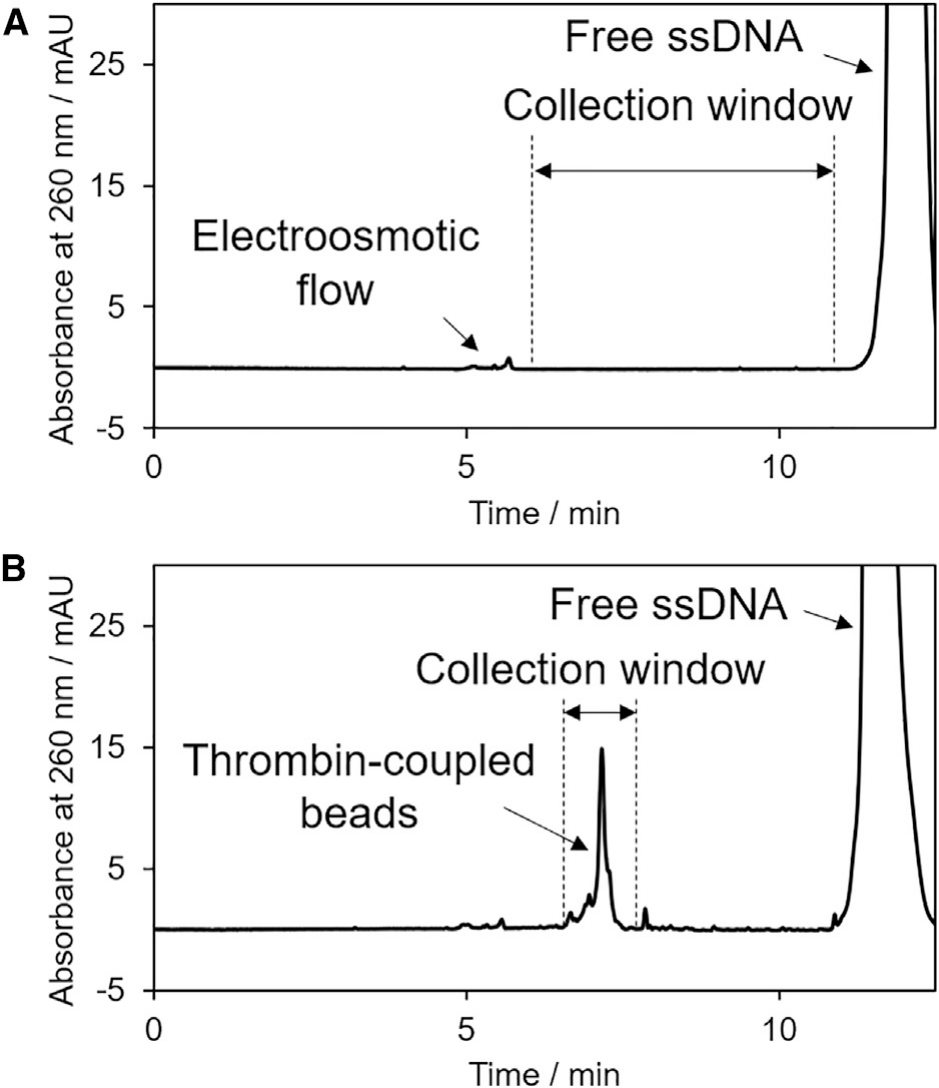
Collection Windows during CE-SELEX and MACE-SELEX (A and B) CE electropherograms for mixtures of (A) a 10 μM ssDNA library and 200 nM thrombin as well as (B) a 10 μM ssDNA library and 200 nM thrombin-coupled beads ([beads] = 0.1 mg/mL = 7–9 × 107 beads/mL); injection volume, 98 nL. The collection windows include the area between the EOF peak and the onset of the ssDNA library peak (6.0–10.9 min) in CE-SELEX and the microbead peak area (6.8–7.8 min) in MACE-SELEX, respectively.

In the MACE-SELEX system, we initially coupled thrombin with microbeads. To inhibit any nonspecific binding of DNA molecules to the bead surface, negatively charged beads possessing carboxylic acid groups were used.^37^ Thrombin was covalently linked to the carboxylic acid groups via formation of an amide bond. We confirmed coupling of thrombin on the bead by a significant CE mobility shift because of the zeta potential shift of the bead surface (Figures S1A and S1B). The motility of the beads changed depending on the immobilized amount of thrombin on the bead surface, and the reproducibility of CE runs was sufficient to estimate the elution time of the beads (Figure S2). Using thrombin-coupled and thrombin-free beads, we examined nonspecific single-stranded DNA (ssDNA) binding to the bead surface. After mixing the ssDNA library with thrombin-coupled or thrombin-free beads, CE fractionation of the ssDNA adsorbed on the beads was carried out (Figures S1C and S1D). As shown in Figure S3, the adsorbed amount of ssDNA on thrombin-coupled beads was significantly higher than that on thrombin-free beads; the PCR product of the non-specifically adsorbed amount of ssDNA on the thrombin-free beads was virtually undetectable. In the CE electropherogram of CE-SELEX, the peak of the free ssDNA was detected at time (*t*) = 11.6 min, whereas thrombin and the thrombin-aptamer complexes were not detectable (Figure 2A). Because the peak of the aptamer and target complexes cannot be detected in the case of CE-SELEX, target-binding aptamers are generally collected during a broad collection window that does not overlap with the peak of free ssDNA. On the other hand, the CE electropherogram of MACE-SELEX showed the peak of the free ssDNA and a peak for the thrombin-coupled beads in the first selection round (Figure 2B). Using light scattering on the beads coupled with the target protein thus was a simple but effective way to detect the aptamer-thrombin complexes on the beads, and all aptamers that strongly bound to the targets on the beads were collected by CE fractionation.

### Sequencing to Identify Aptamer Candidates

After three selection rounds, we employed high-throughput sequencing (HTS) using an Ion Personal Genome Machine (PGM) system to identify the aptamer sequences. As a result, approximately 100,000–1,000,000 sequences were obtained per round, enabling identification of even those candidates that showed merely a slight enrichment. The top ten sequences for CE-SELEX (C01–C10) and MACE-SELEX (M01–M10), abundances, and K_d_ values are summarized in Table 1. In the case of CE-SELEX, the abundance ratio of the most enriched sequence (C01) was 0.16% after three rounds of selection. The abundance ratio of CE-SELEX tends to be low in general because of the heterogeneity of the selected pools in CE-SELEX.^48^ On the other hand, the abundance ratio of M01 was 43% after three rounds, a more than 250-fold enrichment in MACE-SELEX compared with CE-SELEX. The difference in their abundance ratio should be attributed to the difference in their collection window (Figure 2). The broad collection window of CE-SELEX contains the targets, DNA-target complexes, and free DNA molecules that dissociated from the targets during migration in the capillary.^40^ Because the broad collection window in CE-SELEX includes a variety of aptamer candidates with high and low affinity, the abundance ratio tends to be lower than that of MACE-SELEX. In contrast, the narrow collection window in MACE-SELEX contains only aptamers with a strong binding ability because aptamers with a weak binding ability are released during migration in the capillary, leading to enrichment of the aptamer candidates with a higher abundance ratio. As shown in Table 1, the K_d_ values for the aptamers obtained by MACE-SELEX (4.5 ± 0.4 to 231 ± 66 nM) were actually superior to those of CE-SELEX (57 ± 7.3 to 5,278 ± 1,019 nM). K_d_ values largely depend on the instruments and experimental conditions (summarized in Table S1). Thus, for comparison, the K_d_ values of the previously reported aptamers were also measured under the same experimental conditions (Figure S4). HD1 and HD22 are the best known TBAs because of the high affinity and specificity.^15^, ^27^ These two aptamers are commonly used as model aptamer target sets to probe new concepts related to the application of aptamers.^49^ The affinity of the 26-mer aptamer NU172 is known to be higher than that of HD1.^50^ Thr-08 (also called Thrombin 03) is an 100-mer TBA that exhibits 2–3 orders of magnitude higher than those of HD1 and HD22.^51^, ^52^ Interestingly, half of the aptamers obtained by MACE-SELEX (M03, M04, M05, M06, and M08) exhibited a higher affinity than HD22 (13 ± 1.6 nM) or NU172 (12 ± 1.7) and the same order of magnitude as Thr-08 (2.9 ± 0.7 nM).

**Table 1:** Abundance and K_d_ Values (Mean ± SE, n = 3) of the Most Enriched Sequences after Three Selection Rounds

	ID	Sequence	Abundance (%)	K_d_ (nM)
CE-SELEX	C01	5′-AGCAGCACAGAGGTCAGATG**GTTTGGGTGGTTAGGTGTTGACCTGGGATG**CCTATGCGTGCTACCGTGAA-3′	0.16	3577 ± 398
	C02	5′-AGCAGCACAGAGGTCAGATG**GAGTCGGGTGGCTATTGGGTATGGACCGTG**CCTATGCGTGCTACCGTGAA-3′	0.16	5278 ± 1019
	C03	5′-AGCAGCACAGAGGTCAGATG**GATGGTGTAGGTTGGGAGAGGCTCAGTGCC**CCTATGCGTGCTACCGTGAA-3′	0.064	57 ± 7.3
	C04	5′-AGCAGCACAGAGGTCAGATG**TTGGTGGGGTGGCTTTGGGTATTTACTTGG**CCTATGCGTGCTACCGTGAA-3′	0.047	66 ± 1.0
	C05	5′-AGCAGCACAGAGGTCAGATG**GTGGATTTGGGTGGATTGGTATGAACTGAC**CCTATGCGTGCTACCGTGAA-3′	0.043	173 ± 7.6
	C06	5′-AGCAGCACAGAGGTCAGATG**GTTGGGTAGGGTTGGATAGGGGCAAGTAGA**CCTATGCGTGCTACCGTGAA-3′	0.043	63 ± 9.0
	C07	5′-AGCAGCACAGAGGTCAGATG**GTGTACTATTATGGTGTGGTTGGTATGGTT**CCTATGCGTGCTACCGTGAA-3′	0.042	270 ± 55
	C08	5′-AGCAGCACAGAGGTCAGATG**GGTTGGGTGGTGTGGGTAGTGATCCCTGTG**CCTATGCGTGCTACCGTGAA-3′	0.037	102 ± 2.9
	C09	5′-AGCAGCACAGAGGTCAGATG**TGGATTGGTTGGATTGGGGGTGTGACTGTG**CCTATGCGTGCTACCGTGAA-3′	0.033	159 ± 9.2
	C10	5′-AGCAGCACAGAGGTCAGATG**TCGGGTTGGATTGGTTGGCTTAAACTATGT**CCTATGCGTGCTACCGTGAA-3′	0.022	140 ± 7.3
MACE-SELEX	M01	5′-AGCAGCACAGAGGTCAGATG**TTAGGGTTGGGAGGGTGGCTGACTAATGTA**CCTATGCGTGCTACCGTGAA-3′	43	179 ± 23
	M02	5′-AGCAGCACAGAGGTCAGATG**AAGAGGGTGGAGTGGTTGGCTTCACAATGG**CCTATGCGTGCTACCGTGAA-3′	19	124 ± 6.1
	M03	5′-AGCAGCACAGAGGTCAGATG**GTGGTCGGGGTGGTGGGATGAGGGTTCTGA**CCTATGCGTGCTACCGTGAA-3′	7.1	5.4 ± 1.1
	M04	5′-AGCAGCACAGAGGTCAGATG**GCGTGGTAGGGCAGGTTGGGGTCCATGTTG**CCTATGCGTGCTACCGTGAA-3′	4.0	4.5 ± 0.4
	M05	5′-AGCAGCACAGAGGTCAGATG**GCCGTGGTAGGGTAGGTTGGGGTGCCATGA**CCTATGCGTGCTACCGTGAA-3′	2.1	6.3 ± 1.7
	M06	5′-AGCAGCACAGAGGTCAGATG**ATGGAGGTTGGTCGGGTGGGCAATCATTCT**CCTATGCGTGCTACCGTGAA-3′	1.7	8.2 ± 1.5
	M07	5′-AGCAGCACAGAGGTCAGATG**TTAGGGGTTGGGAGGGTGGCTGACTAATGTA**CCTATGCGTGCTACCGTGAA-3′	1.7	231 ± 66
	M08	5′-AGCAGCACAGAGGTCAGATG**ATGGGGATGGGGGGTTGGAGGAATGGATGA**CCTATGCGTGCTACCGTGAA-3′	0.84	7.0 ± 0.6
	M09	5′-AGCAGCACAGAGGTCAGATG**GGGTTGGATTGGGTGGCGGTGTGAACTATG**CCTATGCGTGCTACCGTGAA-3′	0.60	99 ± 16
	M10	5′-AGCAGCACAGAGGTCAGATG**AGCGGGGTTGGGGGGGGTGGAGGAGCTCGTT**CCTATGCGTGCTACCGTGAA-3′	0.54	59 ± 11

Bold text indicates the core region.

The binding selectivity of the obtained aptamers was investigated using a CE-based affinity assay. For this purpose, mixtures of each aptamer and other serum proteins, such as BSA and apo-transferrin, were injected into the CE using a fused silica capillary. The appearance of complex peak patterns and a decrease of the peak associated with unbound DNA were not observed (Figure S5), indicating that all aptamers obtained in this study selectively bind to thrombin.

### Anticoagulant Activity of Aptamers

**Figure 3:**
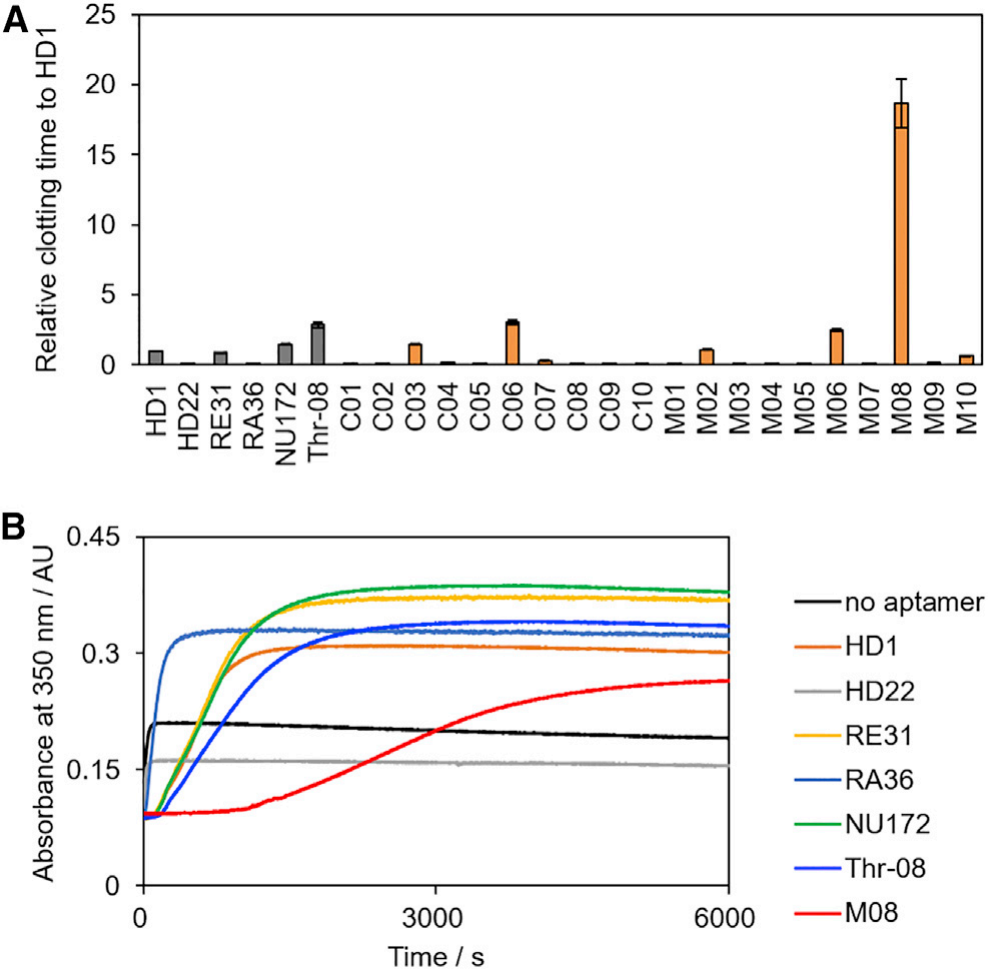
Anticoagulant Activity of Thrombin-Binding Aptamers (A) Comparison of the clotting times of thrombin bound to different aptamers. The clotting time of thrombin bound to HD1 was defined as 1, and the relative values based on it are plotted. (B) Real-time monitoring of light scattering generated by the coagulation process in the presence of M08 and previously reported aptamers. Final concentrations: [aptamer], 500 nM; [thrombin], 50 nM; [fibrinogen], 0.4 mg/mL. Error bars: SE, n = 3.

**Figure 4:**
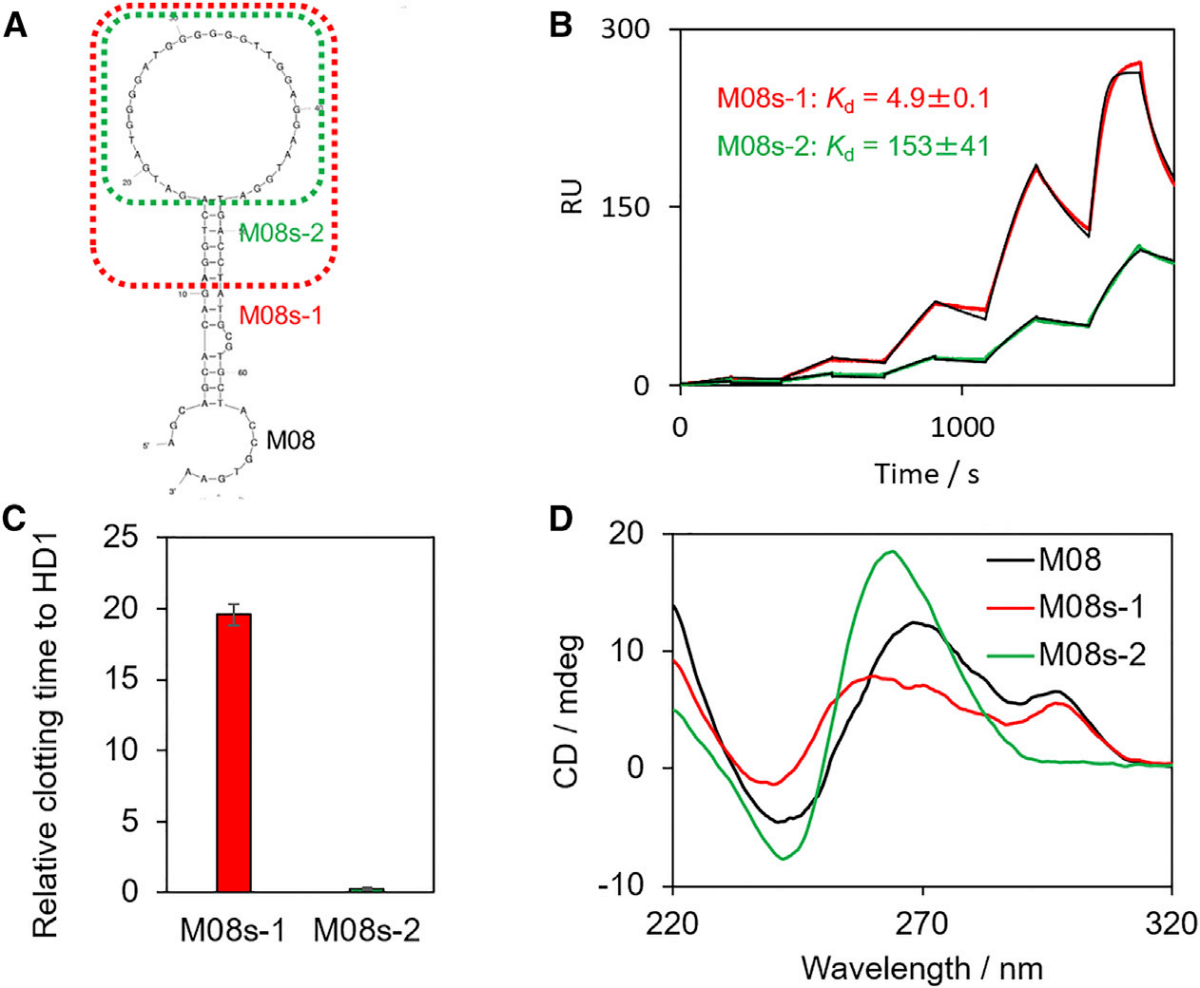
M08 Aptamer Optimization (A) Secondary structure of M08 predicted using mfold. Two types of truncated M08 variants, M08s-1 (43-mer) and M08s-2 (31-mer), were generated. (B–D) SPR sensorgrams (B), anticoagulant activity (C), and CD spectra of M08 and the variants (D), respectively. Error bars: SE, n = 3.

To assess the inhibitory activity of aptamers, a fibrinogen clotting assay was performed. The thrombin-induced formation of an insoluble network, the fibrin gel, can be monitored via light scattering.^53^ To validate the ability of the obtained aptamers, HD1 was used as a control for comparison. HD1 interacts with exosite I and inhibits binding of fibrinogen to thrombin, resulting in anticoagulation.^15^ In addition, the inhibitory activity of several reported aptamers, such as HD22, NU172, RE31, RA36, and Thr-08, was also examined. NU172, RE31, and RA36 are a new generation of thrombin antagonists. As shown in Figure 3A, NU172, RE31, and Thr-08 delayed the coagulation time by about 1.5- to 3-fold compared with HD1, whereas HD22 had a slight inhibition because it interact with exosite II,^54^ which is not a critical domain of fibrinogen degradation. For the same reason, M03–M05, highly homologous with HD22, showed no inhibition. Remarkably, M08 showed a nearly 20-fold longer prolonged clotting time compared with HD1 and significantly surpassed those of other reported aptamers at a 500 nM aptamer concentration (Figure 3B). Even at an aptamer concentration lower than 500 nM, M08 exhibited higher anticoagulant activity compared with the clinically developed DNA aptamers HD1 and NU172 (Figure S6). Next, focusing on this extremely highly anticoagulant aptamer M08, the sequence was optimized to improve affinity and activity. The predicted secondary structure of M08 obtained using the mfold program^55^ was a large hairpin loop with a small bulge loop structure (Figure 4A). Several nucleotides in the loop are critical for binding and are present in all high-affinity aptamers.^56^ Truncation of the bulge loop (M08s-1, 43-mer, 5′-AGG TCA GAT GAT GGG GAT GGG GGG TTG GAG GAA TGG ATG ACC T-3′) did not change the affinity and inhibitory activity, whereas truncation of the all stem (M08s-2, 31-mer, 5′-GAT GAT GGG GAT GGG GGG TTG GAG GAA TGG A-3′) showed a significant decline in performance (Figures 4B and 4C). Because this large loop consists of a G-rich sequence, M08 was predicted to form a duplex and quadruplex mixed structure, similar to the reported TBAs (e. g. HD22, RE31, and NU172).^57^ The topology of G-quadruplex structures is classified into three groups—parallel, mixed or hybrid, and antiparallel—and can be predicted by circular dichroism (CD) spectrum.^58^ As shown in Figure 4D, the CD spectra of M08 and M08s-1 presented positive signals at 295 nm and a broad positive band around 250 to 280 nm. This indicated that M08 and M08s-1 formed an antiparallel or hybrid quadruplex with the duplex. On the other hand, the spectra of M08s-2 showed a positive signal at about 260 nm and a negative one at about 240 nm, which were typical signatures of a parallel quadruplex. These results proved that the stem in M08 was essential for the proper folding of the quadruplex in the large loop to exhibit high affinity and activity.

### Mobility of Thrombin/Aptamer Complexes during CE

**Figure 5:**
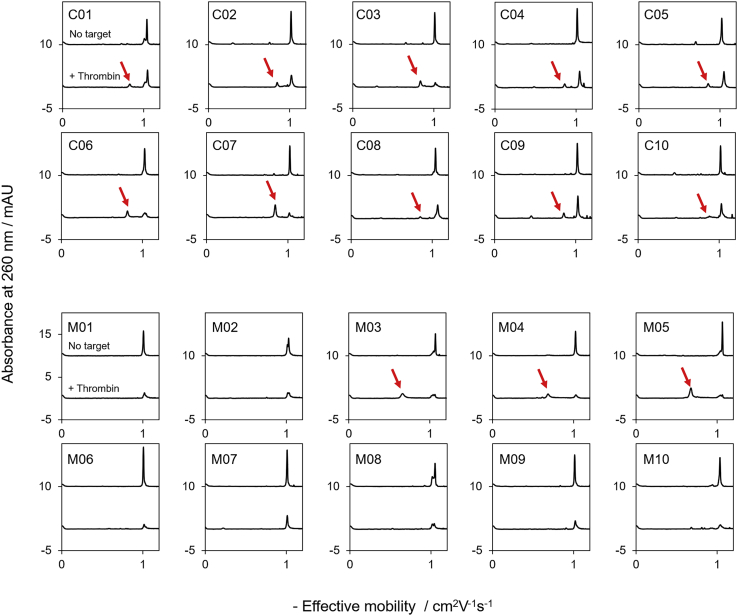
Electropherograms of Aptamer and Thrombin Complexes Obtained from CE Using a Fused Silica Capillary Shown are CE electropherograms of 500 nM aptamers and mixtures of 500 nM aptamers with 1 μM thrombin. Injection volume, 20 nL. Sample buffer: 20 mM Tris-HCl (pH 7.4), 10 mM NaCl, 1 mM MgCl2, and 0.01% Tween 20 (v/v). Separation buffer, 100 mM borate (pH 8.5). Arrows indicate the peaks of complexes, which were estimated based on the absorption spectra.

In addition to the binding strength, anticoagulant activity, and abundance at each round, we found an interesting difference in the CE separation profiles of aptamers obtained from CE-SELEX and MACE-SELEX. Ideally, in the presence of an electro-osmotic flow (EOF), the thrombin-aptamer complex should migrate off the capillary earlier than the free DNA because of its larger size and lower electrophoretic mobility. In the case of C01–C10 and M03–M05, the appearance of aptamer-thrombin complex peaks was observed at earlier migration times than that of the free DNA peak along with a decrease of the free DNA peak area (Figure 5, top). In contrast, no complex peaks were observed for most of the aptamers obtained by MACE-SELEX (M01–M02 and M06–M10) despite the significant decrease of the peak areas for free DNA (Figure 5, bottom). The migration time of the complex peaks might thus be delayed or undetected because of adsorption of the complex onto the capillary surface. To examine this hypothesis, we employed CE separation with a CE phase (CEP)-coated capillary to suppress the interaction between the surface of the fused silica capillary and the aptamer-thrombin complex. As shown in Figure S7, all complex peaks for thrombin with the M01–M02 and M06–M10 aptamers were observed. These analyses of the complexes using both the bare and the coated capillaries strongly support that the aptamer and thrombin complexes consisting of M01–M02 and M06–M10 adhere to the fused silica capillary. In addition, the complexes with M01–10 showed heterologous CE mobility compared with those with C01–10 (Figure S8), suggesting that the complex with various electrophoretic mobility could be obtained by MACE-SELEX because of the dominant mobility of the microbeads. Furthermore, the results of the sequence analysis suggest that M01–M02 and M06–M10 cannot be identified by conventional CE-SELEX. Therefore, large numbers of aptamer candidates with strong binding affinity might have been missed so far using conventional CE-SELEX. In other words, MACE-SELEX enables efficient enrichment of aptamer candidates that bind to targets with high affinity and, presumably, form more hydrophobic complexes than those identified by conventional CE-SELEX.

### Antidote Efficacy of Toehold-Mediated Strand Displacement

**Figure 6:**
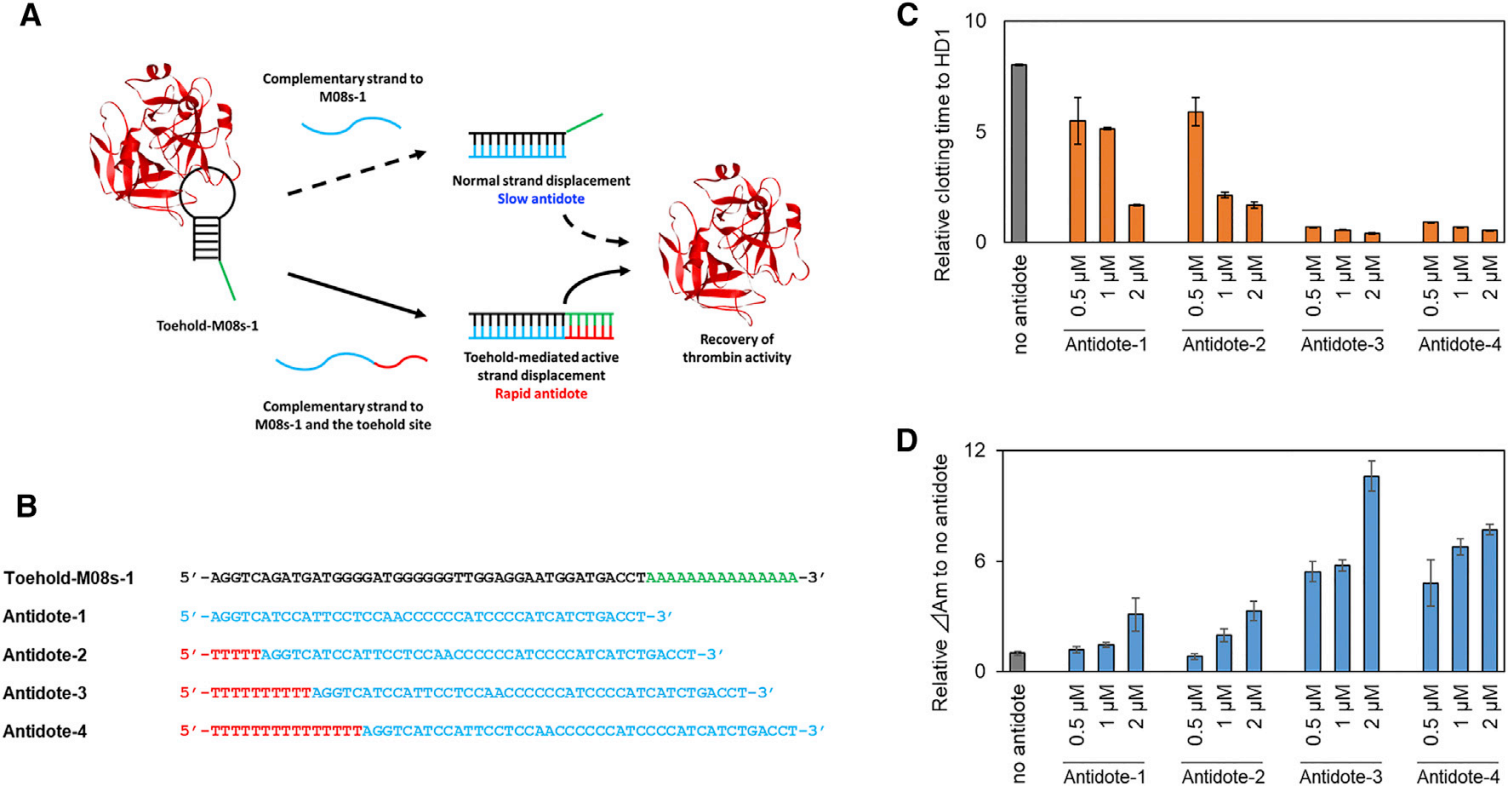
Efficacy of Antidotes by Toehold-Mediated Strand Displacement (A) Schematic illustration of antidotes by normal or toehold-mediated strand displacement with a sequence complementary to the aptamer. (B) Sequences of the toehold-M08s-1 aptamer and the antidote oligonucleotides (antidotes 1–4). Black, aptamer domain; green, toehold domain; blue, complementary sequence with the aptamer domain; red, complementary sequence with the toehold domain. The clotting time of thrombin bound to HD1 was defined as 1, and the relative values based on it are plotted. (C and D) Dose dependency of antidote efficacy. Relative clotting time (C) and ΔΑm (D) to no antidote after adding antibodies with various concentrations and lengths of toehold-complementary sequences. Error bars: SE, n = 3.

To examine the reversible anticoagulant activity of M08s-1, the effect of the full complementary sequence against the aptamer was investigated. The clotting mixture, consisting of thrombin with M08s-1 and fibrinogen, was treated with the antidote and monitored by scattering (Figure S9). Despite the excess amount of the antidote, a gradual scattering increase was observed, taking about 200 s to reach the maximum slope of the clotting curve (ΔAm). This result might be due to the kinetic barrier derived from the equilibrium between rigid aptamer and thrombin complex and the stable duplex of the aptamer. To increase the quenching efficacy of the antidote, toehold-mediated strand exchange was introduced to the antidote system. A short dangling end called a “toehold” is known to drastically accelerate strand exchange^59^, ^60^, ^61^ and could be effective for rapid neutralization of aptamer function. Initially, M08s-1 with an additional 15-nt poly(A) overhang section (Toehold-M08s-1) and several lengths of antidotes were designed (antidotes 1–4), as described in Figures 6A and 6B. Antidote 1 was the complementary sequence with only the aptamer domain. Antidotes 2 to 4 were the complementary sequences with both the aptamer and the toehold domain. As expected, the antidotes with the lowest and highest efficacy were antidote 1 and antidote 3, respectively. With treatment of antidote 1 and antidote 3, the clotting time decreased by 32% to 79% and 91% to 95%, respectively, whereas ΔAm increased by about 1.3-fold to 3.1-fold and 5.4-fold to 11-fold, respectively (Figures 6C and 6D).

**Figure 7:**
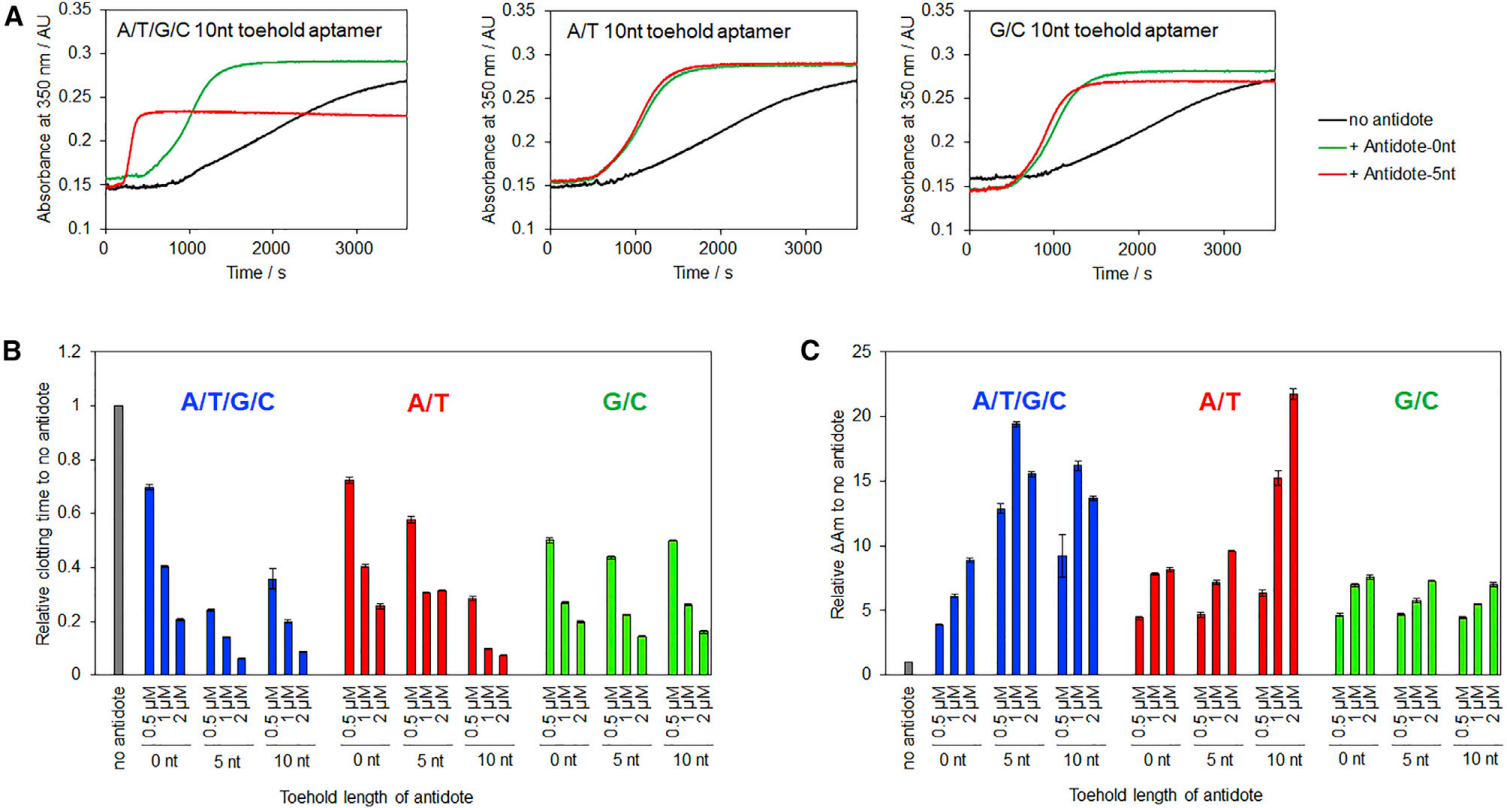
Efficacy of Antidotes against Aptamers with 10-nt Toehold Sequences Consisting of Different A/T/G/C Ratios (A) Real-time monitoring of light scattering generated by the coagulation process in the presence of each aptamer with or without antidotes. Antidotes with 0- and 5-nt toehold-complementary sequences were used. The final concentrations were as follows: aptamer, 0.5 μM; thrombin, 50 nM; fibrinogen, 0.4 mg/mL; antidote, 0.5 μM. (B and C) Relative clotting time (B) and ΔAm (C) to no antidote after adding antidotes with various concentrations and lengths of toehold-complementary sequences. The final concentrations were as follows: aptamer, 0.5 μM; thrombin, 50 nM; fibrinogen, 0.4 mg/mL; antidote, 0.5, 1, and 2 μM. Error bars: SE, n = 3.

To minimize the toehold sequence for reduction of the synthetic cost and redundant effect on the original aptamer, further optimization was performed by using M08s-1 with 10-nt toehold sequences consisting of different A/T/G/C ratios (Table S2). According to a previous report, the G/C-rich toehold sequence exhibits superior efficacy of strand exchange compared with A/T-rich and A/T/G/C mixed sequences because of the formation of a comparably stable duplex.^61^ However, addition of the G/C-rich toehold sequence showed no improvement in antidote efficacy (Figure 7). This is probably because of undesired interactions between the toehold and aptamer or antidote containing G/C-rich domains. Similar to the poly(A) toehold, the antidote with the 10-nt toehold sequence complementary to the A/T toehold drastically improved the antidote activity (Figures 6 and 7). Notably, the A/T/G/C toehold greatly enhanced the antidote efficacy despite the shortest 5-nt toehold complementary sequence; with treatment of equal concentrations of antidotes to the aptamer, the clotting time decreased by 76%, and ΔAm increased by 13-fold relative to no antidote treatment (Figure 7). These results suggest that the A/T/G/C mixed sequence is the most suitable for the toehold-mediated antidote system, probably because of the higher stability of the duplex compared with the A/T sequence and fewer undesired interactions between the toehold and aptamer or antidote compared with the G/C sequence. Finally, the same experiment was carried out using M08s-1 with the 5-nt A/T/G/C toehold sequence and the antidote. As a result, the clotting time decreased by 83% and ΔAm increased by 12-fold relative to no antidote treatment (Figure S10). The case of REG-1 system, where at least a 10-fold higher amount of the antidote than the aptamer was required to suppress approximately 90% of the inhibitory activity,^22^ indicates the superiority of the toehold-mediated antidote system.

## Discussion

In summary, we established the MACE-SELEX strategy for simple and rapid enrichment of high-affinity aptamers. Furthermore, the toehold-mediated rapidly reversible anticoagulant system has been developed using the newly discovered aptamer M08.

We reported previously that CE partitioning is effective to enrich aptamers against cellular microparticles (e.g., bacterial cells and mammalian cells) with synthetic polymer additives.^19^, ^62^ Although MACE-SELEX is similar to these methods in principle, introducing synthetic microparticles to CE-SELEX is the first attempt in the present study. Because MACE separation does not need synthetic polymer additives, the high reproducibility of CE runs was accomplished as shown in Figure S2. Intuitively, introducing a solid support into CE-SELEX seems to be a contradictory approach because non-specific adsorption to a solid support should suppress enrichment of aptamer candidates during SELEX. Actually, CE-SELEX has the advantage of not requiring target molecule immobilization. However, by directly introducing microbeads into CE separation, highly sensitive detection and strict partitioning of the aptamer-target complexes were accomplished. It is important to note that MACE-SELEX is applicable to any type of bead-bound targets with minimal tuning (e.g., measuring the mobility of target-coupled beads), whereas CE-SELEX is limited to targets that exhibit a significant mobility shift upon binding to oligonucleotides.

Notably, the aptamer with extraordinal anticoagulant activity was discovered by MACE-SELEX. The anticoagulant activity of M08 and the 43-mer variant, M08s-1, was 13-fold higher than that of NU172, which has advanced to a phase II clinical trial. Intriguingly, at least 70% of the aptamers obtained from MACE-SELEX, including M08, did not appear in the collection window of conventional CE-SELEX because of the increased interaction with the fused silica capillary upon binding to thrombin. Because of the dominant mobility of the microbeads, aptamer-target complexes with diverse mobility were collected by MACE separation.

In addition, the toehold-mediated antidote was developed. Adding the short 5-nt toehold sequence to the aptamer accomplished rapid neutralization of aptamer activity in a comparably low concentration of antidote. Combining the toehold-mediated antidote and the technology of enhanced blood circulation^63^, ^64^ could facilitate tight regulation of thrombin inhibitors, resulting in more effective and safe antithrombotic therapy. Of course, this rapid antidote system would also be applicable to other aptamers for more cost-effective and safe administration. Finally, the rapidly reversible TBAs developed in the present study could serve as a new candidate for anticoagulant drugs or elements to design novel multivalent aptamers as well as the HD1 and HD22 bivalent aptamers.^24^, ^25^, ^65^

## Materials and Methods

### Chemical Reagents

Thrombin from human plasma and apo-transferrin were purchased from Sigma-Aldrich (USA). BSA and fibrinogen from human plasma were purchased from Wako Pure Chemicals Industries (Japan). All ssDNAs, except for the random ssDNA library with its primer set, were synthesized by Sigma-Aldrich (USA). 2-Morpholinoethanesulfonic acid (MES; Wako, Japan), 1-ethyl-3-(3-dimethylaminopropyl) carbodiimido hydrochloride (EDC; Wako, Japan), Dulbecco’s PBS(−) (Wako, Japan), 10% Tween 20 solution (Bio-Rad, USA), tris(hydroxymethyl)aminomethane (Tris; Wako, Japan), 1 M sodium chloride solution (Wako, Japan), 1 M magnesium chloride solution (Wako, Japan), 1 M sodium hydroxide solution (Agilent Technologies, USA), 0.5 M borate buffer at pH 8.5 ± 0.2 (Polysciences, USA), EDTA disodium salt dihydrate (Wako, Japan), and boric acid (Wako, Japan) were used as received. All solutions were prepared using ultrapure water from a Milli-Q water purification system (Merck Millipore, USA). For the preparation of gels, a 37.5:1 (40%, w/v) acrylamide/bis solution, 2.6% C (Serva Electrophoresis, Germany), ammonium persulphate (Bio-Rad, USA), and *N,N,N’,N’*-tetramethylethylene (TEMED; Bio-Rad, USA) were used. The loading buffer and a 25-bp DNA stepladder were purchased from Wako (Japan).

### Preparation of Thrombin-Coupled Microbeads

Thrombin was coupled with magnetic beads using carboxylic acid groups (Dynabeads MyOne carboxylic acid, 1-μm diameter; Invitrogen, USA) via an amine-coupling reaction according to the manufacturer’s protocol. Briefly, the beads (10 mg/mL, 50 μL) were washed with 10 mM MES buffer (pH 6.0) using a magnet stand. The carboxyl groups were activated with 200 μL of a solution of EDC (10 mg/mL) dissolved in 10 mM MES buffer (pH 6.0) and rotation for 30 min at room temperature. Soon after removing the EDC solution, 200 μL of a thrombin solution (3.2 μM in 10 mM MES buffer; pH 6.0) was added. After incubation with rotation for 3 h at room temperature, the beads were washed with 1 mL of PBS (pH 7.4) that contained 0.1% Tween 20 (v/v) by rotating for 30 min. After removing the PBS, the beads were suspended in 50 μL of CE sample buffer that consisted of 20 mM Tris-HCl (pH 7.4), 10 mM NaCl, 1 mM MgCl_2_, and 0.01% Tween 20 (v/v) as a stock solution. The final concentration of thrombin-coupled beads in the stock solution was 10 mg/mL (7–9 × 10^9^ beads/mL). The amount of coupled thrombin per bead (7–9 × 10^5^ molecules) was determined by measuring the amount of unreacted thrombin in the supernatant using Nanodrop 2000 (Thermo Fisher Scientific, USA). Finally, to confirm the modification of thrombin on the bead surface, 32 nL of thrombin-coupled and thrombin-free bead solution was injected into CE at 1.45 psi for 3 s, and then their zeta potential shifts were compared (Figures S1A and S2B).

### Partitioning of Aptamer Candidates Using CE

Fractionations of aptamers were performed on an Agilent 7100 CE system with a UV detector and a bare fused silica capillary with an extended light path (75 μm inner diameter [i.d.], 365 μm outer diameter [o.d.], 80.5 cm total length, 72 cm effective length; Agilent Technologies, USA). All solutions used for CE were filtered using a 0.22-μm filter (Merck Millipore, USA). Prior to the CE separations, electrodes were washed with DNA-OFF solution (Takara Bio, Japan) to avoid contamination with undesired DNA. For preconditioning, a fused silica capillary was rinsed with 0.1 M NaOH for 10 min and CE separation buffer (100 mM borate buffer, pH 8.5) for 10 min. The ssDNA library containing a central 30-nt random region flanked by two 20-nt primer regions (5′-AGC AGC ACA GAG GTC AGA TG-N30-CCT ATG CGT GCT ACC GTG AA-3′), forward primer (5′-AGC AGC ACA GAG GTC AGA TG-3′), and reverse primer (5′-biotin-CCT CTC TAT GGG CAG TCG GT-3′) were synthesized by Eurofins (Brussels, Belgium). The ssDNA library was dissolved in CE sample buffer, heated to 94°C for 2 min, and allowed to cool slowly (0.1°C/s) to room temperature. Then the ssDNA library was mixed with thrombin or thrombin-coupled beads and incubated for 30 min at room temperature. In the initial selection round, the final concentrations of ssDNA library and thrombin were 10 μM and 100 nM, respectively. In the second and third selection rounds, the injection samples were prepared by mixing the regenerated ssDNA and thrombin or thrombin-coupled beads. During SELEX, the injection volume of the mixtures was 98 nL at 1.45 psi for 9 s. A potential of 30 kV was applied during the separation. The elution time of the aptamer candidates was calculated according to the ratio between the effective and the total capillary length. After each separation, the capillary was rinsed consecutively with DNA-OFF solution, 0.1 M NaOH, and CE separation buffer.

### PCR Amplification and ssDNA Regeneration

The PCR reaction mixtures consisted of 400 μL PrimeSTAR HS (Takara Bio, Japan), 192 μL ultrapure water, and 80 μL 4 μM primers. The mixtures were dispensed into eight PCR tubes, and 6 μL of the collected CE samples were added to each of the six tubes. To the tubes of positive and negative controls, 6 μL of 10 pM ssDNA library and ultrapure water were added, respectively. All PCR tubes were loaded onto a thermal cycler (Takara Bio, Japan), heated to 94°C, and paused for 1 min. After the first denaturation, 23–28 cycles of PCR were performed using 94°C/15 s for denaturation, 55°C/5 s for annealing, and 72°C/20 s for extension. PAGE of the PCR products was performed to observe the desired products. The gels were stained using GelSTAR (Lonza, Switzerland). The PCR products were purified using a Fast Gene PCR product extraction kit (Nippon Genetics, Japan). To generate the ssDNA library for additional rounds of selection, the purified PCR products were separated into single strands using streptavidin-coupled beads (Magnosphere MS300-Streptavidin; Invitrogen, USA). A volume of 5 μL of streptavidin bead solution was used. After washing with binding buffer (20 mM Tris-HCl [pH 7.4], 1 mM EDTA, 2 M NaCl, and 0.1% Tween 20 [v/v]), the beads were mixed with the purified PCR products and incubated for 20 min. Subsequently, the beads were washed several times with binding buffer. The desired ssDNAs were eluted with 0.1 M NaOH and transferred to a dialysis tube (Xpress Micro Dialyzer MD100, molecular weight cut-off [MWCO] 3.5 kDa; Scienova, Germany), which was filled with CE sample buffer to replace the solvent.

### Sequencing

To obtain DNA samples for sequencing, the ssDNA library pools obtained from each round were amplified using fusion primer F1 (5′-CCA TCT CAT CCC TGC GTG TCT CCG ACT CAG-X10-GAT AGC AGC ACA GAG GTC AGA TG-3′; X10 is a bar code sequence to distinguish each sample) and R1 (5′-CCT CTC TAT GGG CAG TCG GTG ATT TCA CGG TAG CAC GCA TAG G-3′), which exhibit the same sequences as the primer set of the ssDNA library with additional adaptor and bar code regions. The sequences of the bar code region used in this study were as follows: X10, 5′-TTC GTG ATT C-3′, 5′-TTC CGA TAA C-3′, 5′-TGA GCG GAA C-3′, and 5′-CTG ACC GAA C-3′. Thermal cycling was performed by incubation at 94°C/1 min, followed by 15 cycles of 94°C/15 s, 66°C/5 s, and 72°C/20 s. After purification with the Fast Gene Gel and PCR Extraction Kit, the PCR products (5′-CCA TCT CAT CCC TGC GTG TCT CCG ACT CAG-X10-GA TAG CAG CAC AGA GGT CAG ATG-N30-CCT ATG CGT GCT ACC GTG AAA TCA CCG ACT GCC CAT AGA GAG G-3′) were obtained. To confirm the purity and concentration of the amplicons, PAGE was carried out. An emulsion PCR was carried out to prepare monoclonal amplicon-attached beads using the Ion OneTouch 2 system (Life Technologies, USA) with a mixture of equal concentration, using each sample as a template. After preparation of the beads, an Ion PGM system (Life Technologies, USA) was employed for sequencing using Ion 314 Chip and 318 Chip for MACE-SELEX and conventional CE-SELEX, respectively. Emulsion PCR, the preparation of beads, and sequencing were performed using the Ion PGM Template OT2 200 Kit, the Ion PGM Sequencing 200 Kit v2, and the Ion 314 (and 318) Chip Kit v2 (Life Technologies, USA) according to the Ion PGM user guidelines (publications MAN0007220 [Rev. 5.0] and MAN0007273 Rev. 3.0]). All sequenced data for each round were exported as FASTQ files and subsequently analyzed using counting tools in the CLC Genomics Workbench (CLC Bio, Denmark) after trimming the primer sequences.

### Measurement of the K_d_ Values of the Aptamers Using SPR

To determine the K_d_ values of the aptamers with thrombin under physiological conditions, binding analyses based on surface plasmon resonance (SPR) spectroscopy were performed using a Biacore X100 (GE Healthcare, UK) at 25°C. For that purpose, thrombin was coupled on a Sensor Chip CM5 (GE Healthcare, UK) by EDC/*N*-hydroxysuccinimide (NHS) chemistry at a flow rate of 10 μL min^−1^ in SPR running buffer (HBS-P, 10 mM HEPES [pH 7.4], 150 mM NaCl, 0.05% [v/v] surfactant P20) (GE Healthcare, UK). Concentration series of aptamers dissolved in SPR running buffer were injected. Basically, we used the mode of multi-cycle kinetics for SPR analysis. However, because aptamers of M06, M08, M10, C06, and Thr-08 were not dissociated by regeneration buffer with high salt concentration (1 M NaCl), we used the mode of single-cycle kinetics for their analyses. In principle, similar K_d_ values can be obtained by both methods.^66^ The obtained data were fitted with a 1:1 binding model using the Biacore X100 evaluation software (GE Healthcare, UK).

### Clotting Time Measurement

To evaluate the anticoagulant activity of each TBA, the clotting time of each sample containing thrombin, each TBA, and fibrinogen substrate in physiological buffer was measured using a microplate reader (Viento Nano; BioTek Japan, Japan). The clotting curve was measured as an increase in absorbance at 350 nm associated with fibrin gel formation. After the annealing process described above, 20 μL each of 10 μM TBA and 20 μL of 1 μM thrombin were added to 280 μL of PBS (pH 7.4) and incubated for 15 min at 25°C. Then, 20 μL of 2 mg/mL fibrinogen was added to each aliquot (80 μL per well) of the reaction mixture. Absorbance recordings were started right after mixing the sample. The final concentrations of thrombin, TBA, and fibrinogen were 50 nM, 500 nM, and 0.4 mg/mL, respectively. In the case of antidote experiments, each antidote molecule was added to the reaction mixture along with fibrinogen. A reaction mixture containing thrombin, HD1, and fibrinogen was always tested together with other samples as an internal standard. All clotting times were normalized based on the internal standard and compared with it. The clotting time and ΔAm were calculated following a procedure described previously.^53^

### CD Measurement

CD spectra of aptamers were obtained with a J-1500 CD spectrometer (JASCO, Japan) using quartz cuvettes with a 1.0-cm path length. The concentrations of all oligonucleotides were 2 μM dissolved in 0.5× PBS (pH 7.4). The measurement was performed at 25°C, and the wavelength ranged from 220 nm to 320 nm. The data gathered were average for five scans at a scanning rate of 100 nm min^−1^ and smoothed using the system software.

### Binding Affinity Assay Using CE with a Fused Silica Capillary

Bulk affinity assays were performed on an Agilent 7100 CE system with a UV detector and a bare fused silica capillary with an extended light path (50 μm i.d., 365 μm o.d., 49.7 cm total length, 41.5 cm effective length; Agilent Technologies, USA). The CE buffers were identical to those used in the selections. Mixtures of 500 nM aptamers with 1 μM of thrombin were incubated for 30 min. A volume of 20 nL of the mixtures was injected into the capillary at 1.45 psi for 6 s. A potential of 30 kV was applied during electrophoresis. As a comparison, electrophoresis of 500 nM of each aptamer in the absence of thrombin was performed. To confirm selectivity, a binding affinity assay to BSA and apo-transferrin was also performed, using the same procedure as that for thrombin.

### Binging Affinity Assay Using CE with a CEP-Coated Capillary

An Agilent 7100 CE system with a laser-induced fluorescence detector (CE-LIF) and CEP-coated capillary (50 μm i.d., 365 μm o.d., 30.2 cm total length, 20 cm effective length, Agilent Technologies, USA) was used for the binding affinity assays. Individual aptamers were labeled with 6-carboxy-fluorescein (6-FAM) at the 5′ end. The CE sample buffer and separation buffer consisted of 10 mM phosphoric acid and 5 mM KCl (pH 7.7). The 100 nM aptamers were mixed with 200 nM of thrombin and kept at room temperature for 30 min. Subsequently, 50 nL of the mixtures was injected into the capillary, and an electric field of −10 kV/cm was applied during the electrophoresis.

## Author Contributions

Conception and Design, K.W. and K.Y.; Acquisition of Data, K.W., A.Y., and M.T.; Analysis and Interpretation of Data, K.W., T.Y., A.Y., M.T., S.S., M.S., H.F., and K.Y.; Writing – Review and/or Revision of the Manuscript, K.W., T.Y., and K.Y.; Administrative, Technical, or Material Support, S.S. and H.F.; Study Supervision, K.Y.

## Conflicts of Interest

The authors (K. W., H. F., and K. Y.,) have filed a patent application (PCT/JP2017/001873, WO2017126646A1).

## Supplemental Information

Document S1. Figures S1–S10 and Tables S1 and S2

Document S2. Article plus Supplemental Information
