# Supplementary material for: Rapidly Neutralizable and Highly Anticoagulant Thrombin-Binding DNA Aptamer Discovered by MACE SELEX
Source: Mol Ther Nucleic Acids. 2019 Mar 22;16:348–59. doi: 10.1016/j.omtn.2019.03.002 (PMC6462803; doi:10.1016/j.omtn.2019.03.002)
Supplement: Document S1. Figures S1–S10 and Tables S1 and S2 [file mmc1.pdf]

OMTN, Volume *16*

Supplemental Information

Rapidly Neutralizable and Highly Anticoagulant Thrombin-Binding DNA Aptamer Discovered by MACE SELEX

Koji Wakui, Toru Yoshitomi, Akane Yamaguchi, Maho Tsuchida, Shingo Saito, Masami Shibukawa, Hitoshi Furusho, and Keitaro Yoshimoto

SUPPLEMENTAL RESULTS

Table S1. Comparison of K_d values of HD1 measured in different experimental conditions.

ID	Method	Buffer	K_d (nM)	Ref.
HD1	SPR (aptamers were immobilized on the chip)	PBS	1.19	1
	Binding assay using nitrocellulose filter	140 mM NaCl, 5 mM KC1, 1 mM $MgCl_2$, 1 mM $CaCl_2$, 20 mM Tris acetate, pH 7.4	75-100	2,3
	Binding assay using microtiter wells	140 mM NaCl, 5 mM KC1, 1 mM $MgCl_2$, 1 mM $CaCl_2$, 20 mM Tris acetate, pH 7.4	1.4-6.2	3,4
	ITC	20 mM Tris–HCl pH 7.4, 140 mM NaCl, 5 mM KCl, 1 mM $MgCl_2$, 1 mM $CaCl_2$	31.25	5
	Capillary electrophoresis	20 mM Tris-HCl, pH 8.3, 5 mM KCl and 1 mM $MgCl_2$	240	6

REFERENCES

(1) Trapaidze, A.; Hérault, J.-P.; Herbert, J.-M.; Bancaud, A.; Gué, A.-M. **2015**.

(2) Macaya, R. F.; Waldron, J. A.; Beutel, B. A.; Gao, H.; Joesten, M. E.; Yang, M.; Patel, R.; Bertelsen, A. H.; Cook, A. F. *Biochemistry* **1995**, *34* (13), 4478.

(3) Tasset, D. M.; Kubik, M. F.; Steiner, W. *J. Mol. Biol.* **1997**, *272* (5), 688.

(4) Tsiang, M.; Gibbs, C. S.; Griffin, L. C.; Dunn, K. E.; Leung, L. L. K. *Journal of Biological Chemistry*. 1995, pp 19370–19376.

(5) Ouellet, E.; Lagally, E. T.; Cheung, K. C.; Haynes, C. A. *Biotechnol. Bioeng.* **2014**, *111* (11), 2265.

(6) Berezovski, M.; Nutiu, R.; Li, Y.; Krylov, S. N. *Anal. Chem.* **2003**, *75* (6), 1382.

Table S2. Sequences of aptamers and antidotes with toehold sequences consisted of different A/T/G/C ratio.

ID	Sequence (5′→3′)
A/T/G/C-Toehold-M08s-1-10nt	AGGTCAGATGATGGGGATGGGGGGTTGGAGGAATGGATGACCTACTGCATGTC
A/T-Toehold-M08s-1-10nt	AGGTCAGATGATGGGGATGGGGGGTTGGAGGAATGGATGACCTATTTATTATA
G/C-Toehold-M08s-1-10nt	AGGTCAGATGATGGGGATGGGGGGTTGGAGGAATGGATGACCTCGCGCCGCCG
A/T/G/C-Toehold-M08s-1-5nt	AGGTCAGATGATGGGGATGGGGGGTTGGAGGAATGGATGACCTACTGC
Antidote-0nt (Antidote-1)	AGGTCATCCATTCCTCCAACCCCCCATCCCCATCATCTGACCT
A/T/G/C-Antidote-5nt	GCAGTAGGTCATCCATTCCTCCAACCCCCCATCCCCATCATCTGACCT
A/T/G/C-Antidote-10nt	GACATGCAGTAGGTCATCCATTCCTCCAACCCCCCATCCCCATCATCTGACCT
G/C-Antidote-5nt	GCGCGAGGTCATCCATTCCTCCAACCCCCCATCCCCATCATCTGACCT
G/C-Antidote-10nt	CGGCGGCGCGAGGTCATCCATTCCTCCAACCCCCCATCCCCATCATCTGACCT
A/T-Antidote-5nt	TAAATAGGTCATCCATTCCTCCAACCCCCCATCCCCATCATCTGACCT
A/T-Antidote-10nt	TATAATAAATAGGTCATCCATTCCTCCAACCCCCCATCCCCATCATCTGACCT

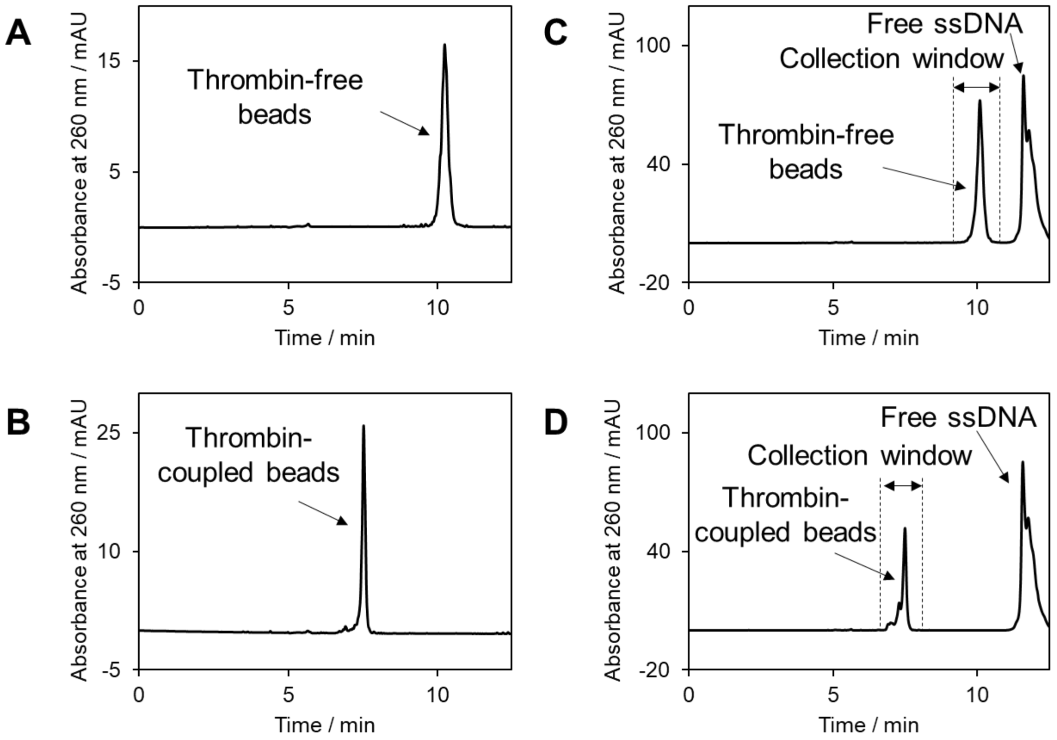

Figure S1. Electropherograms of the beads and the ssDNA library in MACE separation. Injection samples: (A) thrombin-free beads, (B) thrombin-coupled beads, (C) thrombin-free beads with a 10 μM ssDNA library, (D) thrombin-coupled beads with 10 μM ssDNA library; injection volumes: (A, B) 32 nL and (C, D) 65 nL; final concentrations: [beads] = 0.5 mg/mL (3.5-4.5 × 10^8 beads/mL), [protein] = 0.5 μM; sample buffer: 20 mM Tris-HCl (pH = 7.4), 10 mM NaCl, 1 mM MgCl2; separation buffer: 100 mM borate (pH = 8.5).

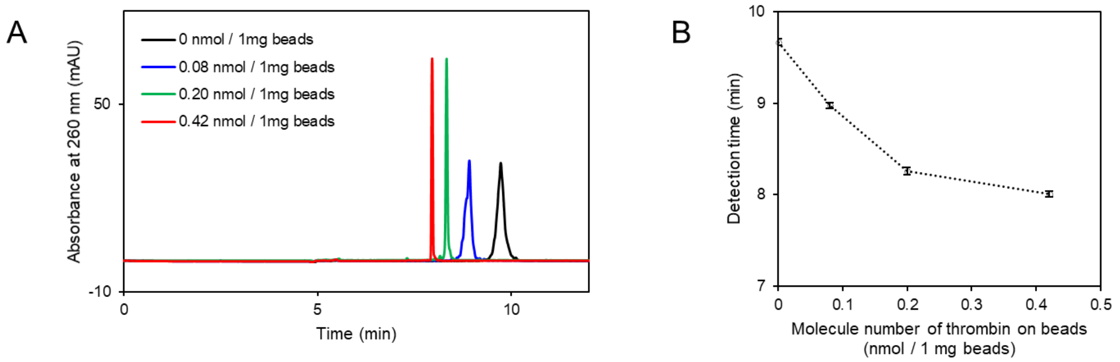

Figure S2. CE mobility shift of microbeads after coupled with thrombin. (A) CE electropherograms of the beads coupled with different concentrations of thrombin. (B) Plots of the detection time of beads. Injection volumes: 32 nL; [beads] = 0.5 mg/mL (3.5-4.5 × 10^8 beads/mL); sample buffer: 20 mM Tris-HCl (pH = 7.4), 10 mM NaCl, 1 mM $MgCl_2$; separation buffer: 100 mM borate (pH = 8.5). The data are expressed as means ± SE; n = 3.

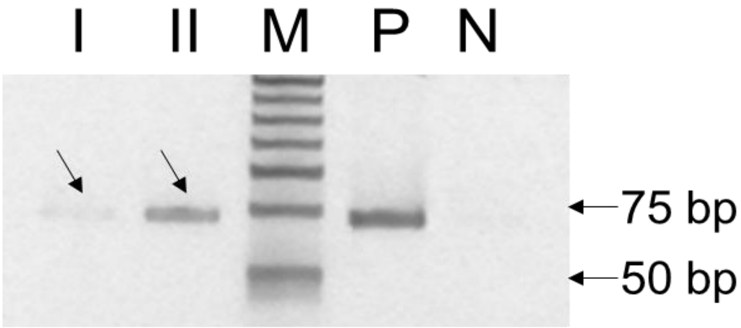

Figure S3**.** Polyacrylamide gel electrophoresis (PAGE) of PCR products using ssDNA samples collected from the MACE separation. Template ssDNAs: (lane I) elution from thrombin-free beads in the collection window shown in Fig. S1C; (lane II) elution from the thrombin-coupled beads in the collection window shown in Fig. S1D; (lane P) 10 pM ssDNA library as a positive control; (lane N) ultra-pure milliQ water as a negative control. Lane M is the DNA stepladder. The arrows in lane I and II indicate the desired PCR products.

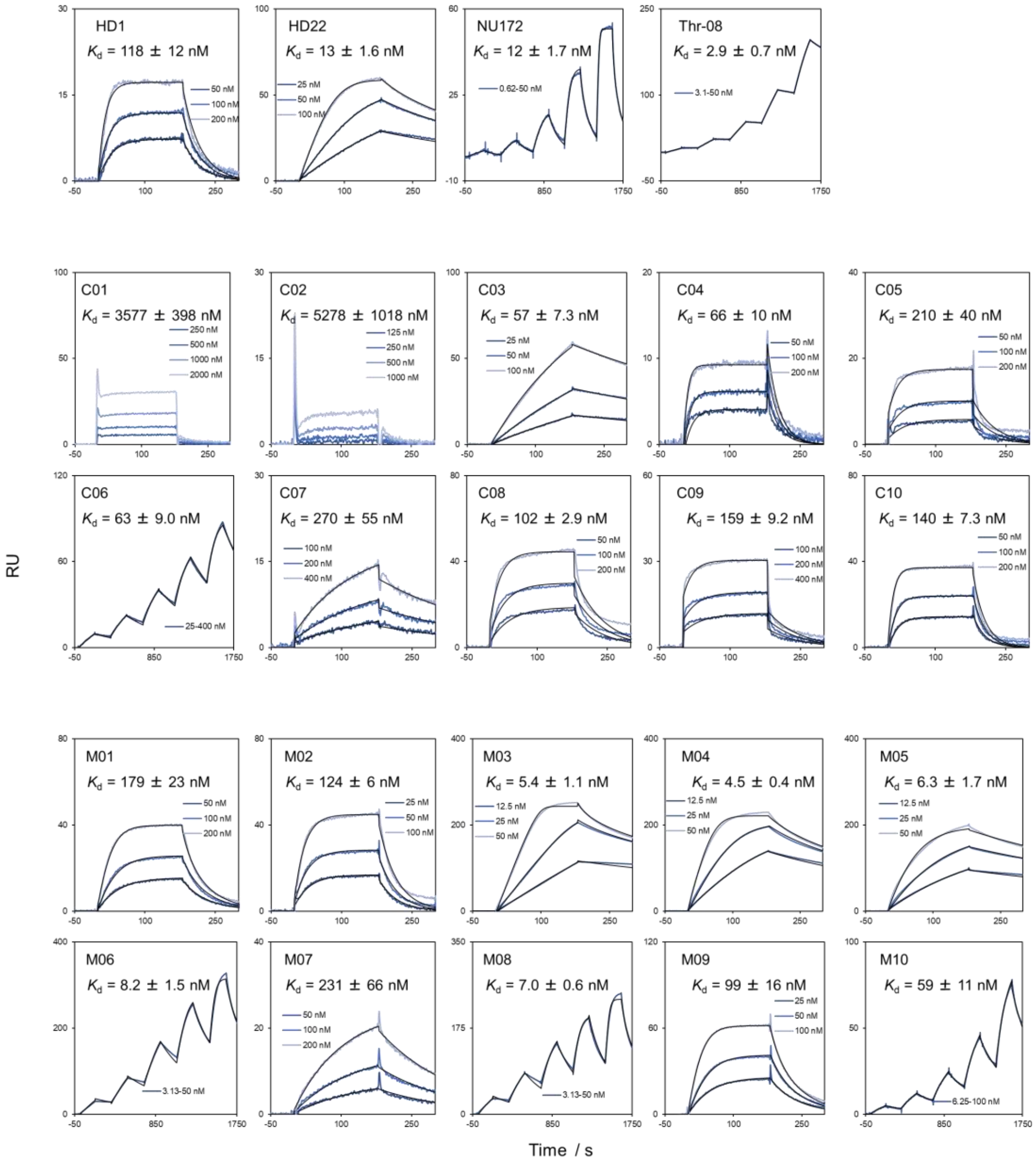

Figure S4. SPR sensorgrams used for estimating K_d (mean ± SE, n = 3) values of the obtained aptamers in HBS-P buffer [10 mM HEPES (pH = 7.4), 150 mM NaCl, 0.05% surfactant P20 (v/v)]. Multi cycle kinetics: HD1, HD22, C01-5, C07-C10. Single cycle kinetics: NU172, The-08, C06, M06, M08, M10.

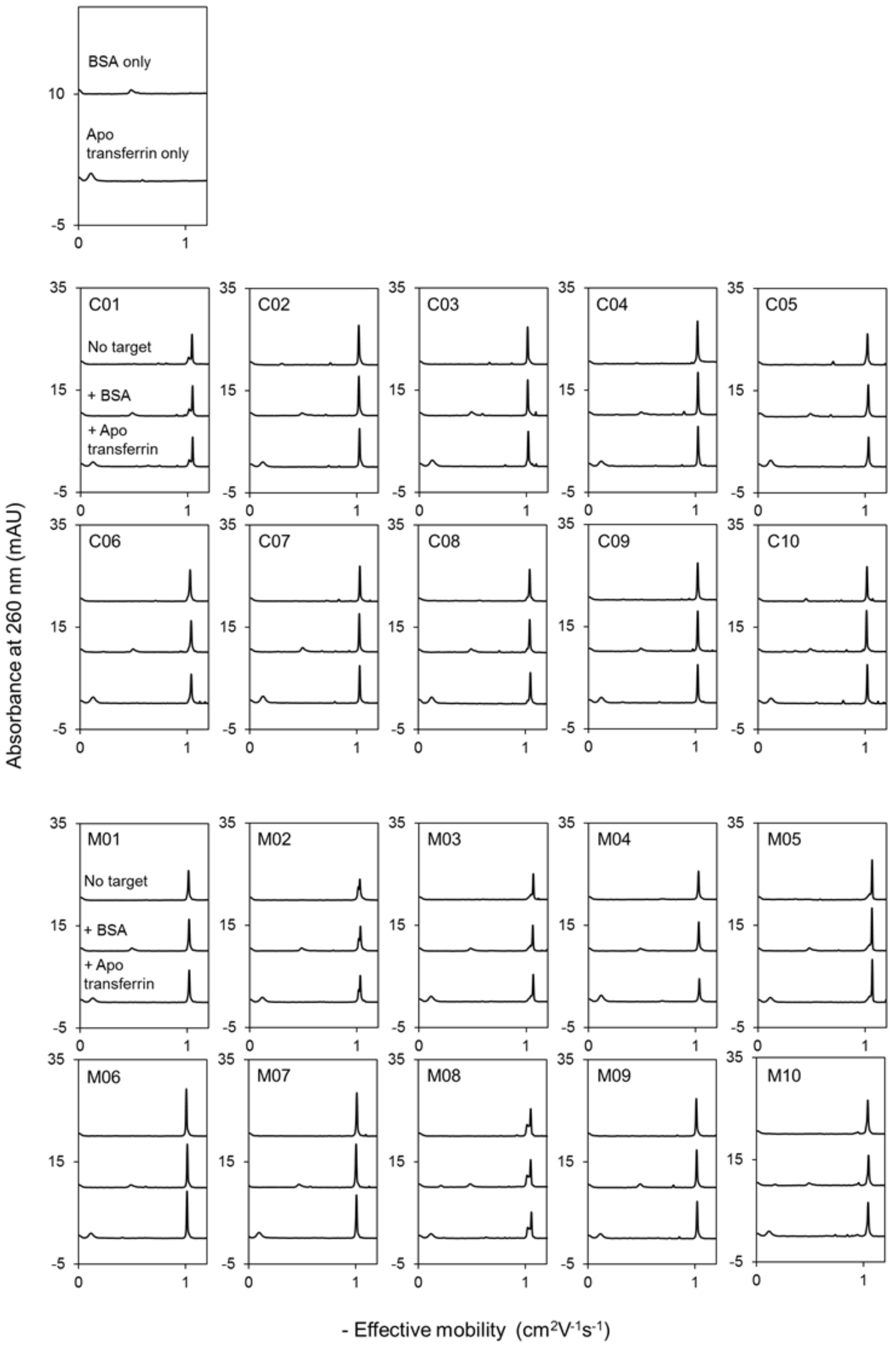

Figure S5 Evaluation of the binding selectivity of the identified aptamers using CE with a fused silica capillary. CE electropherograms of 500 nM aptamers (no target) and the mixtures of 500 nM aptamers with 1 μM BSA (+ BSA) or apo transferrin (+ apo transferrin); injection volume: 20 nL; sample buffer: 20 mM Tris-HCl (pH = 7.4), 10 mM NaCl, 1 mM $MgCl_2$, and 0.01% tween 20 (v/v); separation buffer: 100 mM borate (pH = 8.5).

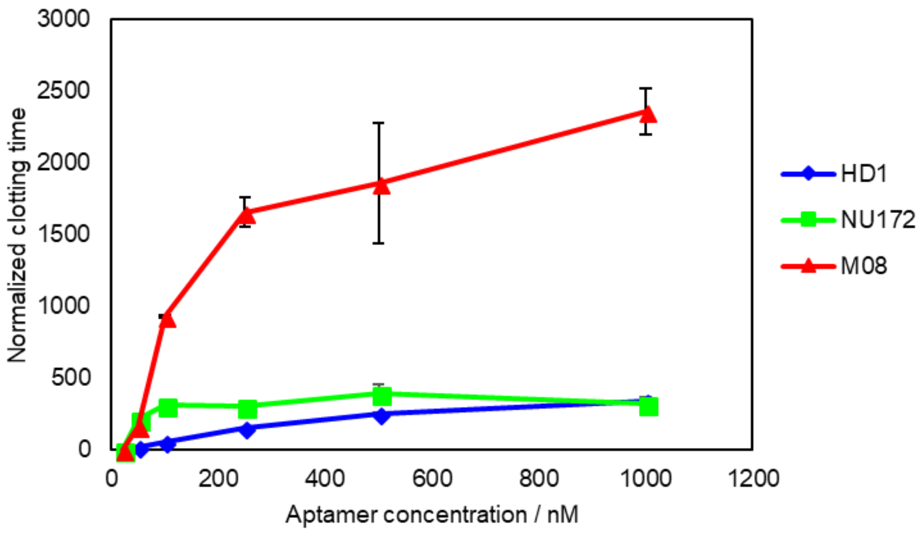

Figure S6. Anticoagulant activity of DNA aptamers with increasing aptamer concentration. Clotting time of thrombin alone was defined as 1, and the relative values of each concentration sample were plotted. The final concentrations: [aptamer] = 20, 50, 100, 250, 500, and 1000 nM, [thrombin] = 20 nM, [fibrinogen] = 0.4 mg/mL.

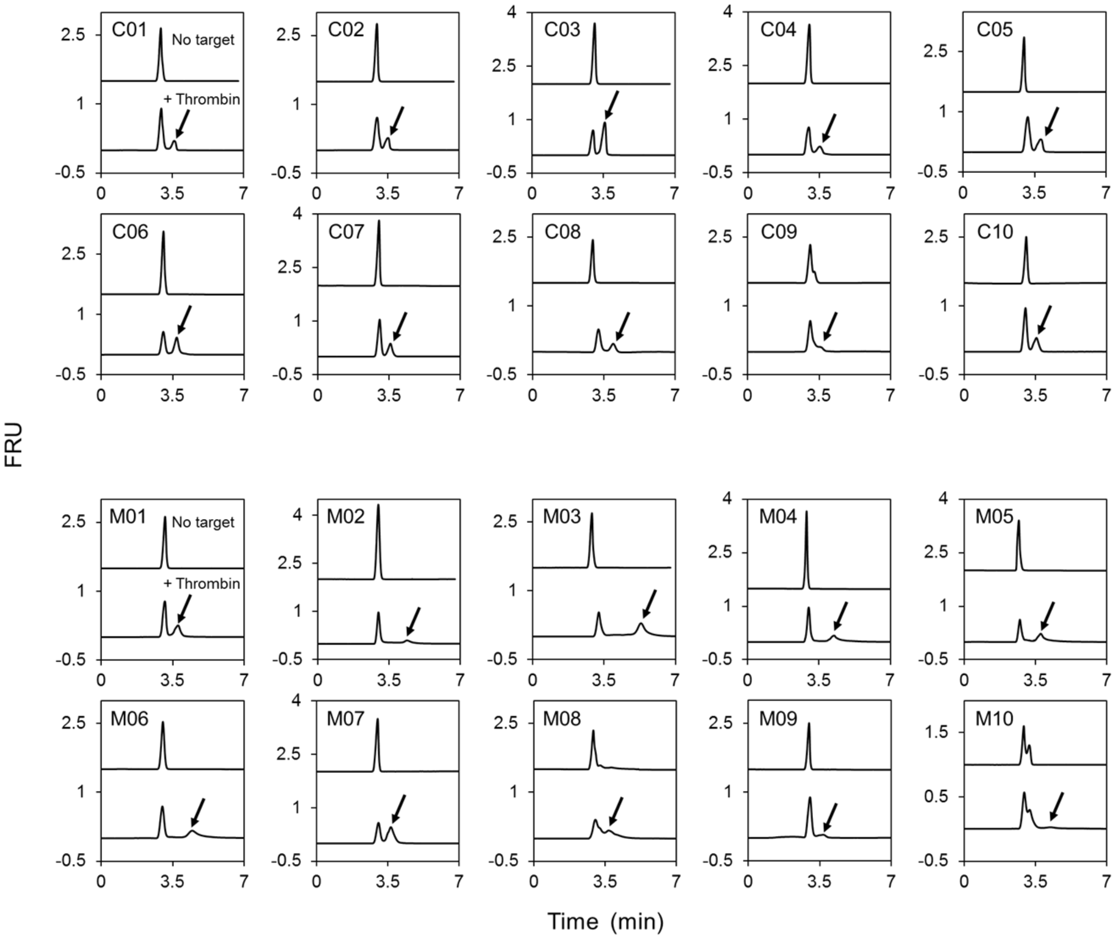

Figure S7. Electropherograms of thrombin-aptamer complexes obtained from CE with a CEP-coated capillary. CE electropherograms of 100 nM aptamers (C01-C10) and the mixtures of 100 nM aptamers and 200 nM thrombin (M01-M10); injection volume: 20-50 nL; sample and separation buffer: 10 mM phosphoric acid, 5 mM KCl (pH = 7.7); arrows indicate the peaks for the complexes.

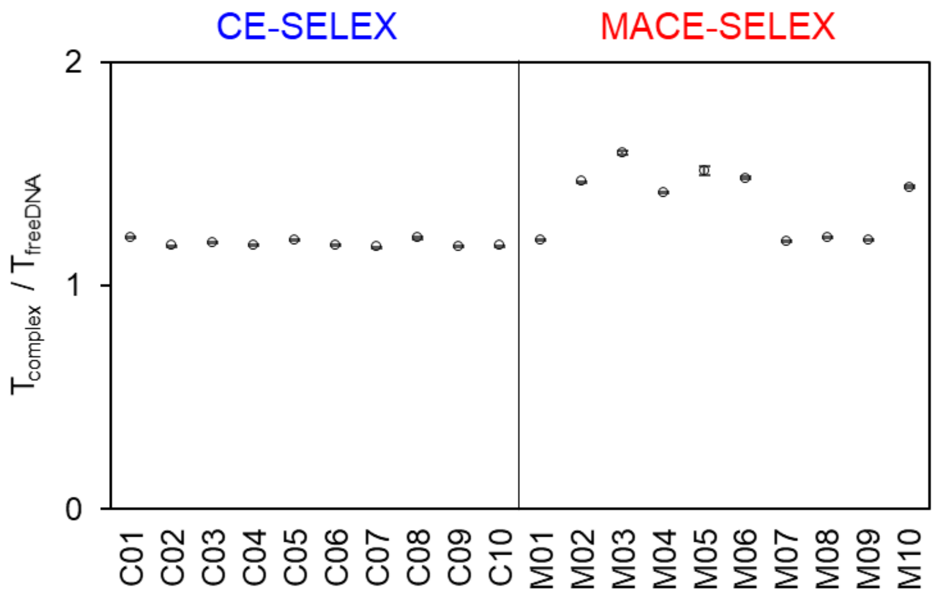

Figure S8. CE mobility of each aptamer/target complex using a CEP-coated capillary. Ratios of the complex and free DNA detection time ($T_{complex}$ / $T_{freeDNA}$) were plotted (n = 3, mean ± SE). Standard deviations (SD) of $T_{complex}$ / $T_{freeDNA}$ of each aptamer group, C01-10 and M01-10, were 0.031 and 0.259 respectively, which were calculated by one-way ANOVA analysis.

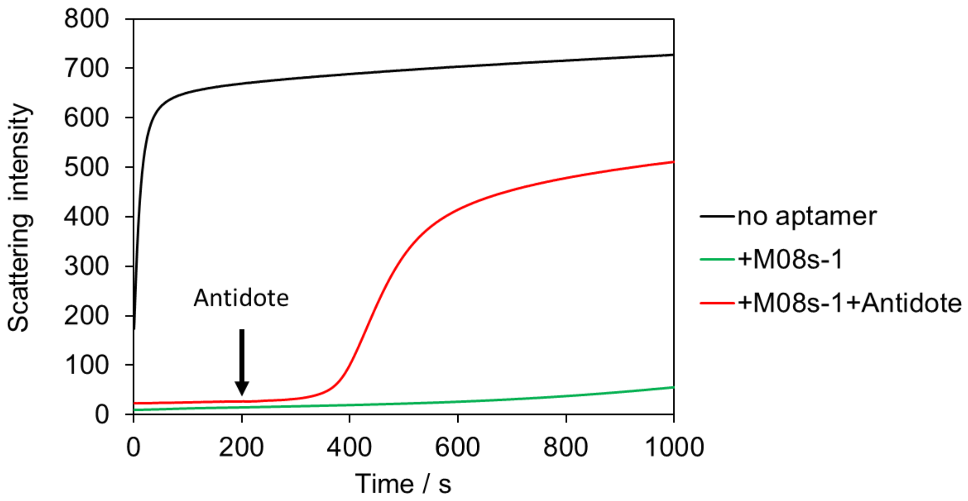

Figure S9. Reversible anticoagulant activity by addition of the antidote oligonucleotide. Black: fibrinogen was added to thrombin at 0 s in the absence of any inhibitors. Red: Antidote (complementary oligonucleotide of M08s-1) was added at 200 s to the mixture of fibrinogen, thrombin, M08s-1. Green: fibrinogen was added to thrombin at 0 sec in the presence of M08s-1. The final concentrations: [fibrinogen]= 0.4 mg/ml, [thrombin]= 50 nM, [aptamer]= 500 nM, [Antidote]= 2.5 μM.

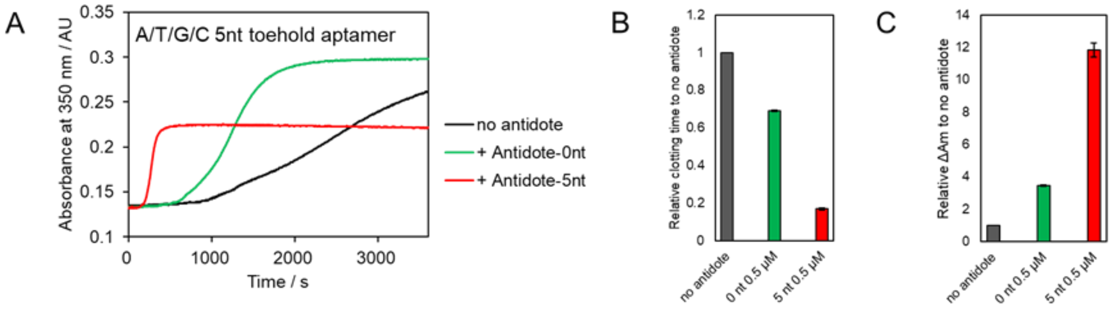

Figure S10. Efficacy of antidotes against M08s-1 with a short A/T/G/C mixed toehold sequence. (A) Real-time monitoring of light scattering generated by the coagulation process in the presence of each aptamers with or without antidotes. Antidotes with 0 and 5 nt toehold complementary sequences were used. (B, C) Relative clotting time and ΔAm to no antidote after adding antidotes with 0 or 5 nt toehold. The final concentration: aptamer = 0.5 μM, thrombin = 50 nM, fibrinogen = 0.4 mg/ml, antidote = 0.5 μM.
